# Education Dominates Modifiable Predictors of Late-Life Cognition: An XGBoost Study

**DOI:** 10.1093/geroni/igaf122.4104

**Published:** 2025-12-31

**Authors:** Jose Cabrero Castro, Eric Klopack, Emma Aguila, Brian Downer, Jennifer Ailshire

**Affiliations:** University of Texas at El Paso, El Paso, Texas, United States; University of Southern California, Los Angeles, California, United States; University of Southern California, Los Angeles, California, United States; University of Texas Medical Branch, Galveston, Texas, United States; University of Southern California, Los Angeles, California, United States

## Abstract

Education, cognitively demanding work, and mentally stimulating leisure activities are theorized to bolster cognitive reserve, but their relative contributions across the life course remain unclear in low and middle income settings. We quantified the effects of education, occupational cognitive complexity, and leisure activities on late life cognition in Mexico, and ranked predictors using Extreme Gradient Boosting (XGBoost). We analyzed cross sectional data from the 2012 Mexican Health and Aging Study including 13,698 adults aged 50 years or older. Global cognition was composite of seven neuropsychological tests. Exposures included years of schooling; occupational cognitive complexity derived by linking longest held occupation to O*NET descriptors; frequency of leisure activities; and current work status. We fit XGBoost with 10 fold cross validation, reported model R squared, and computed gain based feature importance. A secondary model residualized age to isolate modifiable predictors. The full model explained 55 percent of the variance in global cognition (R squared = 0.55). Relative contributions were education 65.4 percent, age 21.1 percent, leisure 9.8 percent, occupational complexity 3.2 percent, and work status 0.5 percent. With age residualized, leisure rose to 21.3 percent and occupational complexity to 10.4 percent. Within leisure, reading contributed the largest gain (11.9 percent), followed by puzzles (3.8 percent) and technology use to communicate with friends (1.7 percent). Schooling was the dominant determinant of late life cognition, but modifiable leisure and occupational characteristics accounted for meaningful additional variation, highlighting opportunities to support cognitive aging in middle income contexts.

